# Determinants of dihydroartemisinin-piperaquine treatment failure in *Plasmodium falciparum* malaria in Cambodia, Thailand, and Vietnam: a prospective clinical, pharmacological, and genetic study

**DOI:** 10.1016/S1473-3099(19)30391-3

**Published:** 2019-09

**Authors:** Rob W van der Pluijm, Mallika Imwong, Nguyen Hoang Chau, Nhu Thi Hoa, Nguyen Thanh Thuy-Nhien, Ngo Viet Thanh, Podjanee Jittamala, Borimas Hanboonkunupakarn, Kitipumi Chutasmit, Chalermpon Saelow, Ratchadaporn Runjarern, Weerayuth Kaewmok, Rupam Tripura, Thomas J Peto, Sovann Yok, Seila Suon, Sokunthea Sreng, Sivanna Mao, Savuth Oun, Sovannary Yen, Chanaki Amaratunga, Dysoley Lek, Rekol Huy, Mehul Dhorda, Kesinee Chotivanich, Elizabeth A Ashley, Mavuto Mukaka, Naomi Waithira, Phaik Yeong Cheah, Richard J Maude, Roberto Amato, Richard D Pearson, Sónia Gonçalves, Christopher G Jacob, William L Hamilton, Rick M Fairhurst, Joel Tarning, Markus Winterberg, Dominic P Kwiatkowski, Sasithon Pukrittayakamee, Tran Tinh Hien, Nicholas PJ Day, Olivo Miotto, Nicholas J White, Arjen M Dondorp

**Affiliations:** a Mahidol Oxford Tropical Medicine Research Unit, Faculty of Tropical Medicine, Mahidol University, Bangkok, Thailand; b Department of Molecular Tropical Medicine and Genetics, Faculty of Tropical Medicine, Mahidol University, Bangkok, Thailand; c Department of Tropical Hygiene, Faculty of Tropical Medicine, Mahidol University, Bangkok, Thailand; d Department of Clinical Tropical Medicine, Faculty of Tropical Medicine, Mahidol University, Bangkok, Thailand; e Centre for Tropical Medicine and Global Health, Nuffield Department of Medicine, University of Oxford, Oxford, United Kingdom; f Oxford University Clinical Research Unit, Hospital for Tropical Diseases, Ho Chi Minh City, Vietnam; g Phu Sing Hospital, Phu Sing, Thailand; h Khun Han Hospital, Khun Han, Thailand; i Pailin Provincial Health Department, Pailin, Cambodia; j National Center for Parasitology, Entomology and Malaria Control, Phnom Penh, Cambodia; k Sampov Meas Referral Hospital, Pursat, Cambodia; l Ratanakiri Referral Hospital, Ratanakiri, Cambodia; m Laboratory of Malaria and Vector Research, National Institute of Allergy and Infectious Diseases, National Institutes of Health, Rockville, MD, USA; n School of Public Health, National Institute of Public Health, Phnom Penh, Cambodia; o WorldWide Antimalarial Resistance Network Asia Regional Centre, Bangkok, Thailand; p Lao-Oxford-Mahosot Hospital Wellcome Trust Research Unit, Vientiane, Laos; q Harvard T H Chan School of Public Health, Harvard University, Boston, MA, USA; r Wellcome Sanger Institute, Hinxton, United Kingdom; s MRC Centre for Genomics and Global Health, Big Data Institute, University of Oxford, Oxford, UK; t The Royal Society of Thailand, Dusit, Bangkok, Thailand

## Abstract

**Background:**

The emergence and spread of resistance in *Plasmodium falciparum* malaria to artemisinin combination therapies in the Greater Mekong subregion poses a major threat to malaria control and elimination. The current study is part of a multi-country, open-label, randomised clinical trial (TRACII, 2015–18) evaluating the efficacy, safety, and tolerability of triple artemisinin combination therapies. A very high rate of treatment failure after treatment with dihydroartemisinin-piperaquine was observed in Thailand, Cambodia, and Vietnam. The immediate public health importance of our findings prompted us to report the efficacy data on dihydroartemisinin-piperaquine and its determinants ahead of the results of the overall trial, which will be published later this year.

**Methods:**

Patients aged between 2 and 65 years presenting with uncomplicated *P falciparum* or mixed species malaria at seven sites in Thailand, Cambodia, and Vietnam were randomly assigned to receive dihydroartemisinin-piperaquine with or without mefloquine, as part of the TRACII trial. The primary outcome was the PCR-corrected efficacy at day 42. Next-generation sequencing was used to assess the prevalence of molecular markers associated with artemisinin resistance (*kelch13* mutations, in particular Cys580Tyr) and piperaquine resistance (*plasmepsin-2* and *plasmepsin-3* amplifications and *crt* mutations). This study is registered with ClinicalTrials.gov, number NCT02453308.

**Findings:**

Between Sept 28, 2015, and Jan 18, 2018, 539 patients with acute *P falciparum* malaria were screened for eligibility, 292 were enrolled, and 140 received dihydroartemisinin-piperaquine. The overall Kaplan-Meier estimate of PCR-corrected efficacy of dihydroartemisinin-piperaquine at day 42 was 50·0% (95% CI 41·1–58·3). PCR-corrected efficacies for individual sites were 12·7% (2·2–33·0) in northeastern Thailand, 38·2% (15·9–60·5) in western Cambodia, 73·4% (57·0–84·3) in Ratanakiri (northeastern Cambodia), and 47·1% (33·5–59·6) in Binh Phuoc (southwestern Vietnam). Treatment failure was associated independently with *plasmepsin2/3* amplification status and four mutations in the *crt* gene (Thr93Ser, His97Tyr, Phe145Ile, and Ile218Phe). Compared with the results of our previous TRACI trial in 2011–13, the prevalence of molecular markers of artemisinin resistance (*kelch13* Cys580Tyr mutations) and piperaquine resistance (*plasmepsin2/3* amplifications and *crt* mutations) has increased substantially in the Greater Mekong subregion in the past decade.

**Interpretation:**

Dihydroartemisinin-piperaquine is not treating malaria effectively across the eastern Greater Mekong subregion. A highly drug-resistant *P falciparum* co-lineage is evolving, acquiring new resistance mechanisms, and spreading. Accelerated elimination of *P falciparum* malaria in this region is needed urgently, to prevent further spread and avoid a potential global health emergency.

**Funding:**

UK Department for International Development, Wellcome Trust, Bill & Melinda Gates Foundation, Medical Research Council, and National Institutes of Health.

## Introduction

In the 1990s, widespread multidrug resistance to *Plasmodium falciparum* malaria throughout southeast Asia led to the introduction of highly effective artemisinin combination therapies (ACTs)^1^. These drugs have since contributed substantially to the sharp decline in the global malaria disease burden and saved millions of lives.^2^ These gains are now threatened by the emergence and spread of artemisinin resistance in southeast Asia.^3^, ^4^ Artemisinin resistance in *P falciparum* has emerged and spread throughout the Greater Mekong subregion, and is characterised by slow in vivo parasite clearance resulting from reduced drug susceptibility of ring-stage parasites.^5^, ^6^, ^7^ Loss of artemisinin efficacy in ACTs has facilitated the re-emergence of mefloquine resistance on the Thailand-Myanmar border and contributed to the emergence and spread of piperaquine resistance in *P falciparum* in Cambodia and southern Vietnam.^8^, ^9^, ^10^, ^11^

**Research in context**

**Evidence before this study**

We searched PubMed for articles published before March 28, 2019, using the terms “artemisinin”, “piperaquine”, “resistance”, and “PfCRT”', in combination with either “Cambodia”, “Vietnam”, or “Thailand”. Of the 80 articles found, 31 articles documented the efficacy of dihydroartemisinin-piperaquine in Cambodia, Vietnam, or Thailand, or documented the prevalence of in vitro phenotypes or genetic markers of artemisinin or piperaquine resistance. Several earlier studies documented good efficacy of dihydroartemisinin-piperaquine throughout Cambodia; however, a study between 2008 and 2010 in western Cambodia found a PCR-corrected efficacy of 75·0% in Pailin and 89·3% in Pursat at day 42. More recent studies between 2012 and 2013 reported a PCR-corrected efficacy of 63·2% in Pursat and 98·4% in Ratanakiri, northeastern Cambodia, at day 63. In Binh Phuoc, southwestern Vietnam, the most recent PCR-corrected efficacy of dihydroartemisinin-piperaquine was reported in 2015 as 65·0% by day 42. A 2012–13 study in Ratanakiri, Pursat, and Preah Vihear in Cambodia found no correlation between treatment outcomes and day 7 piperaquine levels, and baseline parasite densities and patient age, but found that *kelch13* gene mutations and high piperaquine in vitro 50% inhibitory concentrations were predictors of treatment failure. Additionally, amplification in the genes encoding *plasmepsin-2* and *plasmepsin-3* has been associated with in vitro piperaquine resistance and in vivo treatment failure. Imwong and colleagues in 2017 and Amato and colleagues in 2018 independently showed that a parasite co-lineage containing a *kelch13* Cys580Tyr mutation and amplification of *plasmepsin2/3* emerged in western Cambodia and then spread across northeastern Thailand, northern and northeastern Cambodia, and southwestern Vietnam between 2007 and 2015. Recent in vitro gene editing experiments by Ross and colleagues in 2018 using the *Plasmodium falciparum* Dd2 strain and field strains from Cambodia have suggested that multiple mutations (His97Tyr, Phe145Ile, Met343Leu, and Gly353Val) in the *crt* gene also reduce piperaquine susceptibility. The *crt* Phe145Ile mutation has been associated with dihydroartemisinin-piperaquine treatment failure in a study by Agrawal and colleagues in 2017, also after adjusting for the *plasmepsin2/3* amplification status.

**Added value of this study**

This study shows that the efficacy of dihydroartemisinin-piperaquine in the treatment of *P falciparum* malaria has declined substantially in western and northeastern Cambodia, northeastern Thailand, and southwestern Vietnam. The *P falciparum* co-lineage resistant to dihydroartemisinin-piperaquine can now be found throughout the Greater Mekong subregion, and it has acquired mutations in the *crt* gene which are independently associated with higher rates of treatment failure.

**Implications of all the available evidence**

This study reinforces the urgency for accelerated elimination of malaria in the Greater Mekong subregion. Dihydroartemisinin-piperaquine should no longer be used for the treatment of *P falciparum* malaria in the eastern Greater Mekong subregion, since it provides ineffective treatment and thereby contributes to increased malaria transmission. Continued action to prevent the spread of this *P falciparum* multidrug-resistant co-lineage to other parts of Asia and sub-Saharan Africa is urgently needed.

Several non-synonymous mutations in the propeller domain of the kelch13 gene have been associated with artemisinin resistance.^4^, ^12^ Amplification of the *plasmepsin-2* and *plasmepsin-3* genes on chromosome 14 has been strongly associated with reduced in vitro susceptibility to piperaquine, and with reduced treatment efficacy of dihydroartemisinin-piperaquine for uncomplicated *P falciparum* malaria.^13^, ^14^ Genetic epidemiology studies^15^, ^16^ have shown that a haplotype containing the *kelch13* Cys580Tyr (C580Y) mutation (referred to as KEL1 when identified by single nucleotide polymorphism haplotyping and *Pf*Pailin when identified by microsatellite haplotyping) and a haplotype containing a *plasmepsin2/3* amplification (referred to as PLA1) have merged to form a successful multidrug-resistant co-lineage that has spread throughout the eastern Greater Mekong subregion. Recently, mutations in the *crt* gene have been suggested as contributors to piperaquine resistance.^17^, ^18^

The current study is part of a multi-country, open-label, randomised clinical trial (TRACII, 2015–18; registered with Clinicaltrials.gov, number NCT02453308) evaluating the efficacy, safety, and tolerability of triple ACTs in areas with multidrug-resistant *P falciparum* malaria. TRACII recruited patients in 18 regions, across eight countries. In Laos, Bangladesh, India, the Democratic Republic of the Congo, and Myanmar patients were randomly assigned to receive either artemether-lumefantrine and amodiaquine (the triple ACT) or artemether-lumefantrine only. In Cambodia, Vietnam, Thailand, and Myanmar, patients were randomly assigned to receive either dihydroartemisinin-piperaquine and mefloquine (the triple ACT) or dihydroartemisinin-piperaquine only. A very high rate of treatment failure (50·0% [95% CI 41·7–58·9%]) within the first 42 days after treatment with dihydroartemisinin-piperaquine was observed in 140 patients in Thailand, Cambodia, and Vietnam. The immediate public health importance of our findings prompted us to report the efficacy data on dihydroartemisinin-piperaquine and its determinants ahead of the results of the overall trial. Additionally, we report on the evolution of molecular markers for antimalarial drug resistance in the period from the preceding study (TRACI, 2011–13) to the present study.^4^

## Methods

### Study design and participants

The study design for this prospective clinical, pharmacological, and genetic study was adapted from the WHO recommendations for surveillance of antimalarial drug efficacy.^19^ The study took place at seven sites in Thailand, Cambodia, and Vietnam. Patients aged between 2 and 65 years presenting with uncomplicated *P falciparum* or mixed species malaria, parasitaemia on microscopy but below 200 000 parasites per μL, and a tympanic temperature above 37·5°C or a history of fever in the previous 24 h were eligible for inclusion in the study. Written informed consent was obtained from all patients. Exclusion criteria were severe malaria or other severe illnesses necessitating treatment, haematocrit below 25%, allergy or contraindication to the study drugs, use of any artemisinin-containing drug in the previous 7 days or use of mefloquine in the previous 2 months, splenectomy, pregnancy, breast feeding, a QTc interval above 450 ms, or a history of cardiac conduction problems.

### Procedures

After clinical examination and a standardised symptom questionnaire, blood was taken for quantitation of parasitaemia, plasma piperaquine concentrations, and parasite genotyping. Patients were randomly assigned to receive dihydroartemisinin-piperaquine with or without mefloquine using sequentially numbered opaque envelopes, as part of the TRACII trial. Dihydroartemisinin-piperaquine (with or without mefloquine) was administered at baseline and hour 24 and 48 after enrolment. The target dose per day was 4 mg/kg (range 2–10) of dihydroartemisinin and 18 mg/kg (16–27) of piperaquine for adults and children who weighed at least 25 kg, and 4 mg/kg (2·5–10) of dihydroartemisinin and 24 mg/kg (20–32) of piperaquine for children who weighed less than 25 kg. The target dose of mefloquine was 8 mg/kg (5·7–11·4) per day. A single dose of primaquine (0.25 mg base/kg) was administered to all patients at hour 24. Doses were chosen according to the weight-based WHO dosing recommendations (appendix pp 11–14).^20^ All treatments were directly observed. Full doses were re-administered if patients vomited within 30 minutes of receiving the drugs, half doses were re-administered if patients vomited within 30–60 minutes of receiving the drugs. Patients were monitored in hospital for at least 3 days after treatment initiation and were followed up on day 7, and thereafter weekly until day 42. Each follow-up visit included a standardised symptom questionnaire and physical examination and measurement of the tympanic temperature. Patients were encouraged to return to the study centres if new symptoms appeared between follow-up visits. Recurrent *P falciparum* malaria infections were treated with artesunate and atovaquone-proguanil for 3 days in Cambodia and Thailand,^21^ and with quinine and doxycycline for 7 days in Vietnam (appendix pp 11–14).

### Outcomes

The primary outcome for this study was the PCR-corrected efficacy by day 42. We expressed efficacy as recrudescence-free survival estimates using Kaplan-Meier analyses. The results of the overall TRACII trial will be published later this year.

### Laboratory analyses

Parasite densities were quantitated in Giemsa-stained blood smears at baseline and hour 4, 6, 8, and 12 after treatment initiation and every 6 h thereafter until two consecutive thick blood smears were negative (in 200 high-powered fields). Blood smears were repeated at each follow-up. In case of recurrent *P falciparum* malaria infections, blood samples were obtained for parasite genotyping and drug concentration measurements. Venous plasma samples for the measurement of piperaquine concentrations were obtained at baseline and day 7. Plasma piperaquine concentrations were measured using liquid chromatography-tandem mass spectrometry as described previously with a lower limit of detection of 1·2 ng/mL.^22^

DNA for next-generation sequencing of the parasite genotype was extracted from two types of samples collected at the time of admission: 20 μL dried blood spots on filter paper for targeted genotyping by amplicon sequencing (AmpSeq); and 2 mL leucocyte-depleted venous blood, filtered through cellulose column filters (cellulose powder type B, Advantec, Japan) for whole-genome sequencing (WGS). For AmpSeq, DNA from dried blood spots underwent selective whole genome amplification,^23^ and selected primers were used to amplify parasite DNA at the desired loci before sequencing (appendix p 2). Sequence data for both genotyping methods were generated with Illumina short-read technology. WGS read counts were used to call genotypes with a standardised analysis pipeline (Pf6.0 release).^24^, ^25^ WGS samples were genotyped at 1 043 334 quality-filtered coding single nucleotide polymorphisms, identified by the MalariaGEN *P falciparum* Community Project V6.0 pipeline. Genotypes were called with a coverage of at least five reads, and alleles were disregarded when represented by fewer than two reads (or 5% of reads when coverage was >50). To genotype *kelch13*, we scanned sequencing reads from AmpSeq or WGS that aligned to amino acids distal to position 350 in this gene, identifying any nonsynonymous variants. If no such variants were found, the sample was considered wild type. If at least 50% of positions had insufficient coverage the genotype was considered undetermined. A mutation was labelled as heterozygous if a proportion of reads for the wild-type allele were also found at the mutation site. *Plasmepsin2/3* status was assessed by scanning sequencing reads from AmpSeq or WGS for the characteristic duplication breakpoint.^26^ To optimise completeness of genotypic data, we combined multiple methods when applicable. *Kelch13* was genotyped by WGS, supplemented by AmpSeq calls when WGS calls were undetermined. Amplifications of *plasmepsin2/3* and *mdr1* (a marker of mefloquine sensitivity) were quantified using Taqman real-time PCR (rtPCR) following previously described protocols, and supplemented by AmpSeq when rtPCR could not determine *plasmepsin2/3* status.^13^, ^27^ *crt* genotypes were assessed from WGS, except for low-coverage positions 218 and 220, which were assessed from AmpSeq data; remaining missing genotypes were supplemented by PCR assays.^18^ All genotypes for TRACI samples were derived from WGS data. Recurrent infections were classified as recrudescences if all *msp1, msp2*, and *glurp* alleles matched those present at baseline as described previously.^28^

### Statistical analysis

Estimates of recrudescence-free survival on day 28 and 42 after treatment initiation were obtained using Kaplan-Meier analyses. Contributors to treatment failure were assessed using Cox regression (unadjusted and adjusted), using previously described predictors of treatment failure. Patients were censored in the Kaplan-Meier analysis at the day of the following events: loss to follow-up, discontinuation of study drug, withdrawal of consent, discontinuation from the study due to non-compliance to the study protocol, *Plasmodium vivax* infection, PCR-confirmed *P falciparum* reinfection, or PCR-undetermined recurrent *P falciparum* infection. Parasite clearance parameters were estimated using the WorldWide Antimalarial Resistance Network parasite clearance estimator.^29^ For comparisons between regions and other subgroups we used Wilcoxon rank-sum tests or Kruskal-Wallis tests (continuous data) or Fisher's exact test (binomial data). All analyses were done with Stata (version 15.1, Stata Corporation, USA).

### Role of the funding source

The funders of the study had no role in study design, data collection, data analysis, data interpretation, or writing of the report. The corresponding author had full access to all the data in the study and had final responsibility for the decision to submit for publication.

## Results

Between Sept 28, 2015, and Jan 18, 2018, 539 patients with acute *P falciparum* malaria were screened for eligibility, 292 were enrolled, and 140 received dihydroartemisinin-piperaquine (appendix p 4). These 140 patients were recruited in two sites in northeastern Thailand, Phusing (n=15) and Khun Han (n=four); three sites in Cambodia, Pailin (n=nine), Pursat (n=eight), and Ratanakiri (n=44); and two sites in southwestern Vietnam, both in Binh Phuoc province (n=60). Pursat and Pailin are referred to as western Cambodia and Phusing and Khun Han are referred to as northeastern Thailand.

Two patients deteriorated to develop severe malaria. One 45-year-old woman (infected by a parasite with *kelch13* C580Y and *crt* His97Tyr [H97Y] mutations but with no *plasmepsin2/3* amplification) was started on intravenous artesunate 18 h after enrolment, resulting in a rapid recovery. One 49-year-old man (*kelch13* mutation unknown, plasmepsin non-amplified, Ile218Phe [I218F] *crt* mutation) developed a rash after the first dose of dihydroartemisinin-piperaquine, which was interpreted as a drug allergy. As the patient was also deteriorating clinically (respiratory distress), he was started on intravenous quinine and ceftriaxone 12 h after enrolment. Despite an initial clinical recovery and a reduction of parasitaemia, the patient developed fever, hypoxia, and hypotension on day 4. The chest X-ray showed bilateral infiltration. Despite ventilatory support, the patient died the same day. The cause of death was recorded as acute respiratory distress syndrome. No autopsy was done. Another patient had a vasovagal collapse related to a venipuncture. The treating physician changed the antimalarial treatment to intravenous artesunate. Four other patients were censored from the Kaplan-Meier analysis: one because of prolongation of the QTc interval, two because of non-adherence to the study protocol, and one because of loss to follow-up.

118 (84%) of 140 patients treated with dihydroartemisinin-piperaquine were men (table 1). The median age of all patients was 27·0 years (IQR 18·5–37·6). Parasite counts were similar across sites; presence of gametocytaemia, at baseline, assessed by microscopy, ranged between 11·8% in northeastern Thailand and 41·2% in western Cambodia. Five (4%) of 140 patients presented with a *P vivax* co-infection. Piperaquine was detected in baseline blood samples in 30 (23%) of 131 patients. Detectable piperaquine at baseline was associated with admission gametocytaemia (crude odds ratio [OR] 6·05, 95% CI 2·36–15·54; p=0·0001).

**Table 1:** Baseline characteristics of the study population

		**All sites (n=140)**	**Northeastern Thailand (n=19)**	**Cambodia (n=61)**		**Vietnam, Binh Phuoc (n=60)**
				Western Cambodia (n=17)	Ratanakiri (n=44)	
Sex						
	Male	118 (84%)	19 (100%)	17 (100%)	31 (70%)	51 (85%)
	Female	22 (16%)	0	0	13 (30%)	9 (15%)
Age (years)		27·0 (18·5–37·6)	39·1 (29·9–49·2)	29·0 (27·0–38·0)	23 (16·0–35·0)	22 (16·9–33·2)
Patients with fever at baseline >37·5°C		83 (59%)	15 (79%)	5/17 (29%)	29 (66%)	34/60 (57%)
Body temperature at baseline (°C)		37·9 (1·1)	38·7 (1·2)	37·3 (1·0)	37·9 (0·9)	37·8 (1·1)
Weight (kg)		52·6 (13·1)	64·2 (9·4)	59·1 (5·7)	46·6 (11·6)	51·2 (13·7)
Haematocrit (%)		41·0 (4·7)	43·5 (5·5)	40·2 (3·4)	41·8 (4·8)	40·0 (4·6)
Geometric mean parasite count per μL (range)		25 732 (160–214 223)	21 031 (3472–214 223)	16 125 (384–152 604)	17 345 (160–117 562)	41 814 (5024–198 950)
Baseline gametocytaemia		27/137 (20%)	2/17 (12%)	7 (41%)	7/43 (16%)	11 (18%)
Geometric mean gametocyte count per μL (range)		59 (16–5120)	406 (224–736)	95 (16–5120)	51 (16–432)	33 (16–320)
Presence of *Plasmodium vivax* co-infection at baseline		5 (4%)	1 (5%)	1 (6%)	0	3/60 (5%)
Baseline detectable piperaquine		30/131 (23%)	2/18 (11%)	14 (82%)	6 (14%)	8/52 (15%)
Baseline piperaquine plasma concentration (ng/mL)		9·7 (3·5–16·4)	10·7 (9·7–11·6)	6·7 (3·5–12·3)	3·4 (2·1–4·9)	20·3 (11·4–56·0)

Data are n (%), median (IQR), or mean (SD) unless otherwise specified. The baseline parasitaemia of one patient was above the screening cut-off (214 223 parasites per μL) as the parasitaemia rose between screening and baseline.

Parasites were detected by microscopy in 94 (68%) of 138 of patients 72 h after treatment initiation, but before day 7 microscopic parasitaemia cleared in all patients. Parasite clearance half-life (PC_1/2_) could be assessed in 133 patients: 109 (82%) of 133 had a PC_1/2_ of more than 5·5 h (table 2).^30^ Genotyping of the *kelch13* gene found 124 (91%) of 137 samples to contain either C580Y mutations or mixed C580Y and wild-type mutations (table 2). The KEL1 haplotype was found in 104 (88%) of 118 samples. *Plasmepsin2/3* amplification was detected in 103 (74%) of 139 samples, while *mdr1* amplification was not observed. One of the following six *crt* mutations were observed in 92 (74%) of 124 patients: Thr93Ser (T93S), H97Y, Phe145Ile (F145I), I218F, Met343Ile, and Gly353Val (G353V).

**Table 2:** Parasite clearance parameters and molecular markers of antimalarial drug resistance

	**All sites (n=140)**	**Northeastern Thailand (n=19)**	**Cambodia (n=61)**		**Vietnam, Binh Phuoc (n=60)**
			Western Cambodia (n=17)	Ratanakiri (n=44)	
PC_1/2_ (h)	7·0 (1·9)	8·1 (1·5)	5·9 (1·7)	7·2 (2·0)	6·8 (1·8)
PC_1/2_ >5·5 h (%; 95% CI)	109/133 (82%; 74·4–88·1)	18/18 (100%; 81·5–100·0)	10 (59%; 32·9–81·6)	36/42 (86%; 71·5–94·6)	45/56 (80%; 67·6–89·8)
Time to 50% parasite clearance (h)	10·8 (6·5–13·0)	11·7 (10·1–13·2)	10·9 (8·1–12·5)	9·0 (4·8–13·4)	10·5 (6·4–12·7)
Positive blood smear for asexual parasitaemia 72 h after treatment initiation (%; 95% CI)	91/134 (68%; 59·3–75·7)	16/18 (89%; 65·3–98·6)	11 (65%; 38·3–85·8)	22/43 (51%; 35·5–66·7)	42/56 (75%; 61·6–85·6)
*kelch13* Cys580Tyr mutations or mixed infections containing Cys580Tyr mutations (%; 95% CI)	124/137 (91%; 84·3–94·9)	18/18 (100%; 81·5–100·0)	15/16 (93%; 69·8–99·8)	36/43 (84%; 69·3–93·2)	55 (92%; 81·6–97·2)
KEL1 Cys580Tyr mutations or mixed infections containing KEL1 Cys580Tyr mutations (%; 95% CI)	104/118 (88%; 80·9–93·4)	14/14 (100%; 76·8–100·0)	14/16 (88%; 61·7–98·4)	32/38 (84%; 68·7–94·0)	44/50 (88%; 75·7–95·5)
*plasmepsin 2/3* amplification (%; 95% CI)	103/139 (74%; 66·0–81·2)	15 (79%; 54·4–94·0)	11 (65%; 38·3–85·8)	31/43 (72%; 56·3–84·7)	46 (77%; 64·0–86·6)
*mdr1* amplification (%; 95% CI)	0/139 (0%; 0–2·6)	0 (0%; 0–17·6)	0 (0%; 0–19·5)	0/43 (0%; 0–8·2)	0 (0%; 0–6·0)
*crt* Thr93Ser mutation (%; 95% CI)	31/124 (25%; 17·7–33·6)	0/18 (0%; 0–18·5)	1/13 (8%; 0·2–36·0)	6/39 (15%; 5·9–30·5)	24/54 (44%; 30·9–58·6)
*crt* His97Tyr mutation (%; 95% CI)	15/124 (12%; 6·9–19·2)	4/18 (22%; 6·4–47·6)	6/13 (46%; 19·2–74·9)	5/39 (13%; 4·3–27·4)	0/54 (0%; 0–6·6)
*crt* Phe145Ile mutation (%; 95% CI)	16/124 (13%; 7·6–20·1)	2/18 (11%; 1·4–34·7)	0/13 (0%; 0–24·7)	4/39 (10%; 2·9–24·2)	10/54 (19%; 9·3–31·4)
*crt* Ile218Phe mutation (%; 95% CI)	25/124 (20%; 13·5–28·3)	8/18 (44%; 21·5–69·2)	1/13 (8%; 0·2–36·0)	4/39 (10%; 2·9–24·2)	12/54 (22%; 12·0–35·6)
*crt* Met343Ile mutation (%; 95% CI)	2/124 (2%; 0·2–5·7)	0/18 (0%; 0–18·5)	1/13 (8%; 0·2–36·0)	0/39 (0%; 0–9·0)	0/54 (0%; 0–6·6)
*crt* Gly353Val mutation (%; 95% CI)	3/124 (3%; 0·5–6·9)	2/18 (11%; 1·4–34·7)	1/13 (8%; 0·2–36·0)	0/39 (0%; 0–9·0)	0/54 (0%; 0–6·6)

Data are mean (SD) or median (IQR) unless otherwise specified. PC_1/2_=parasite clearance half-life.

**Figure 1:**
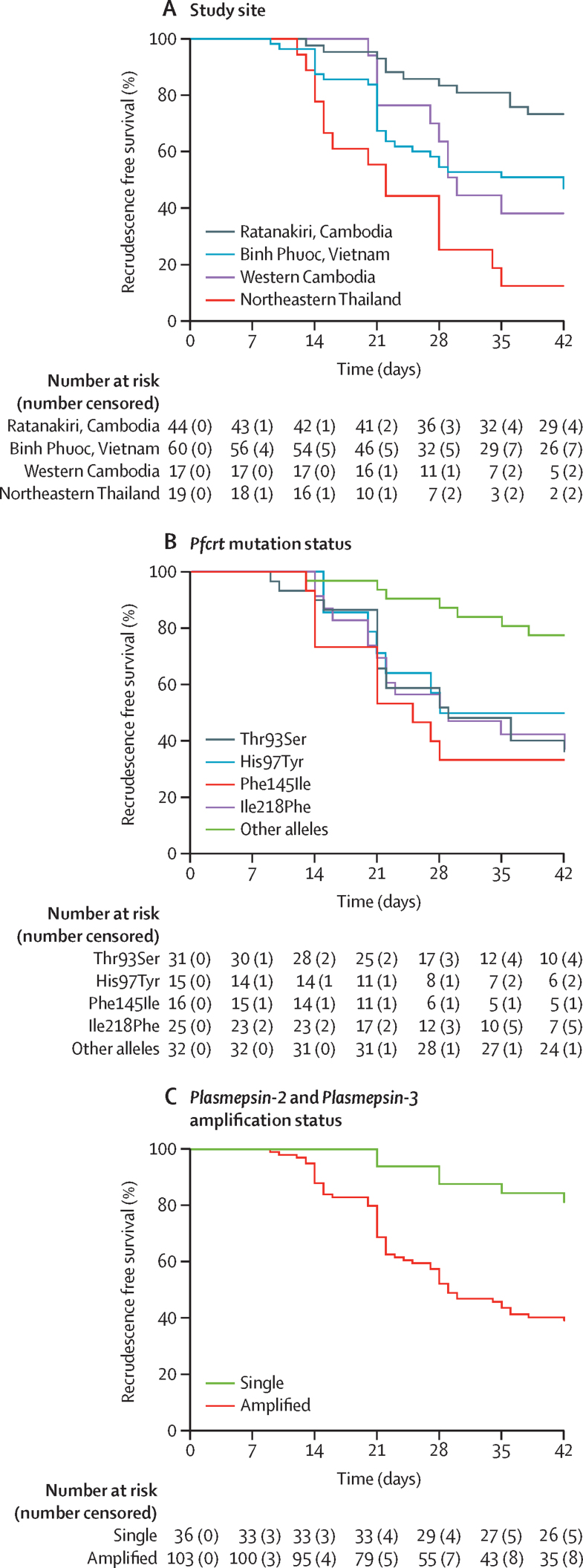
Kaplan-Meier survival curves of PCR-corrected efficacy of dihydroartemisin-piperaquine by (A) study site, (B) *crt* mutation status, and (C) *plasmepsin-2 and plasmepsin-3* amplification status Other *crt* alleles indicate parasites carrying no mutations at positions 93, 97, 145, 218, 343, and 353 of the *crt* gene. Infections caused by parasites with a Met343Ile or Gly353Val *crt* mutation were too scarce to be included in survival curves by *crt* mutation status.

The total follow-up time for the 140 patients was 4270 days (median follow-up was 35 days). The overall Kaplan-Meier estimate of PCR-corrected efficacy of dihydroartemisinin-piperaquine at day 42 was 50·0% (95% CI 41·1–58·3; table 3). PCR-corrected efficacies at day 42 for individual sites were 12·7% (2·2–33·0) in northeastern Thailand, 38·2% (15·9–60·5) in western Cambodia, 73·4% (57·0–84·3) in Ratanakiri (northeastern Cambodia), and 47·1% (33·5–59·6) in Binh Phuoc (southwestern Vietnam; table 3; figure 1). Six (9%) of 71 recurrent infections were classified as reinfections. PCR correction was not possible for two recurrent infections. One patient presented with a *P vivax* infection at day 35. Recrudescent *P falciparum* infections were detected between day 9 and 42, with a median of 21 days. Gametocytes were detected by microscopy in only one of 65 recrudescent infections. There was no difference in the time to recrudescence between study sites (p=0·127; table 3).

**Table 3:** Clinical outcomes related to the efficacy of dihydroartemisinin-piperaquine in the study population

	**All sites (n=140)**	**Northeastern Thailand (n=19)**	**Cambodia (n=61)**		**Vietnam, Binh Phuoc**	**p value**
			Western Cambodia (n=17)	Ratanakiri (n=44)		
Kaplan-Meier estimates of PCR-corrected efficacy on day 42 (95% CI)	50·0% (41·1–58·3)	12·7% (2·2–33·0)	38·2% (15·9–60·5)	73·4% (57·0–84·3)	47·1% (33·5–59·6)	0·0001
Kaplan-Meier estimates of PCR corrected efficacy on day 28 (95% CI)	61·3% (52·5–69·1)	25·4% (8·2–47·2)	63·7% (36·3–81·9)	83·5% (68·4–91·8)	54·7% (40·7–66·7)	0·0009
Patients with fever >37·5°C on day of recrudescent infection (%; 95% CI)	7/65 (11%; 4·4–20·9)	4/15 (27%; 7·8–55·1)	0/10 (0%; 0–30·8)	1/11 (9%; 0·2–41·3)	2/29 (7%; 0·8–22·8)	0·164
Median days from treatment initiation to recrudescent *Plasmodium falciparum* infection (range)	21 (9–42)	20 (12–35)	28 (20–35)	24 (13–38)	21 (9–42)	0·127
Patients with gametocytes visible through microscopy on day of recrudescent infection (%; 95% CI)	1/65 (2%; 0·0–8·3)	1/15 (7%; 0·2–31·9)	0/10 (0%; 0–30·8)	0/11 (0%; 0–21·8)	0/29 (0%; 0–11·9)	0·343
Mean parasite count per μL on day of recrudescence (range)	366 (16–31 651)	224 (16–30 898)	155 (16–4736)	1023 (368–7159)	444 (16–31 651)	0·092
Patients with plasma piperaquine concentration <30 ng/mL at day 7 (%; 95% CI)	52/123 (42%;33·4–51·5)	7/18 (39%;17·3–64·3)	6/16 (38%; 15·2–64·6)	23/43 (54%;37·7–68·8)	16/46 (35%; 21·4–50·2)	0·328
Plasma piperaquine concentration at day 7 (ng/mL)	33·5 (22·8–46·2)	33·0 (23·5–41·8)	34·9 (21·7–44·2)	29·0 (22·0–40·5)	37·7 (25·2–53·8)	0·258
Plasma piperaquine concentration at day of recrudescence (ng/mL)	16·3 (9·4–33·0)	21·3 (15·8–41·9)	13·5 (8·1–30·9)	7·6 (6·4–9·0)	22·1 (14·0–43·5)	0·001

Data are median (IQR) unless otherwise specified. p values are for differences between all four sites and were calculated using either Fisher's exact test (binomial data) or Kruskal-Wallis test (continuous data).

In the unadjusted Cox regression analysis, site of recruitment, *plasmepsin2/3* amplification status, PC_1/2_, and the presence of the *crt* mutations T93S, H97Y, Phe145I and I218F were all associated with treatment failure (figure 1; appendix p 1). Baseline parasitaemias and day 7 plasma piperaquine concentrations (as continuous variable or binary variable with a cut-off of 30 ng/mL)^31^ were not associated with subsequent treatment failure (appendix p 5).

We constructed a multivariable model using three genetic markers: *kelch13* mutation status, *crt* mutation status, and *plasmepsin2/3* amplification status. In this model, *plasmepsin2/3* amplification status and the four *crt* mutations were independently associated with treatment failure. When the site of recruitment (a potential confounder) was added to the model, two *crt* mutations (T93S and F145I) and *plasmepsin2/3* amplification status remained independently associated with treatment failure. *Kelch13* mutation status was not found to be associated with treatment failure both in the unadjusted analysis (HR 3·863 [95% CI 0·943–15·816]; p=0·060) and adjusted analysis (HR 0·632 [95% CI 0·125–3·208]; p=0·580), likely related to the high overall proportion of infections carrying the C580Y *kelch13* mutation. A subgroup analysis of infections carrying both *kelch13* C580Y mutations and *plasmepsin2/3* amplifications showed that *crt* mutations at position 93, 145, and 218 were all associated with reduced clinical efficacy of dihydroartemisinin-piperaquine, suggesting a contribution in addition to the effect of the other two markers (appendix p 6). Within the subgroup of *plasmepsin2/3* amplified parasites, *crt* mutations were not associated with higher plasma piperaquine concentrations at recrudescence, or with the interval to recrudescence (appendix p 7). However, in patients with measurable plasma piperaquine at baseline, the *crt* F145I mutation was associated with higher piperaquine concentrations at baseline (p=0·034) and at day 7 (p=0·023) (appendix p 8).

**Figure 2:**
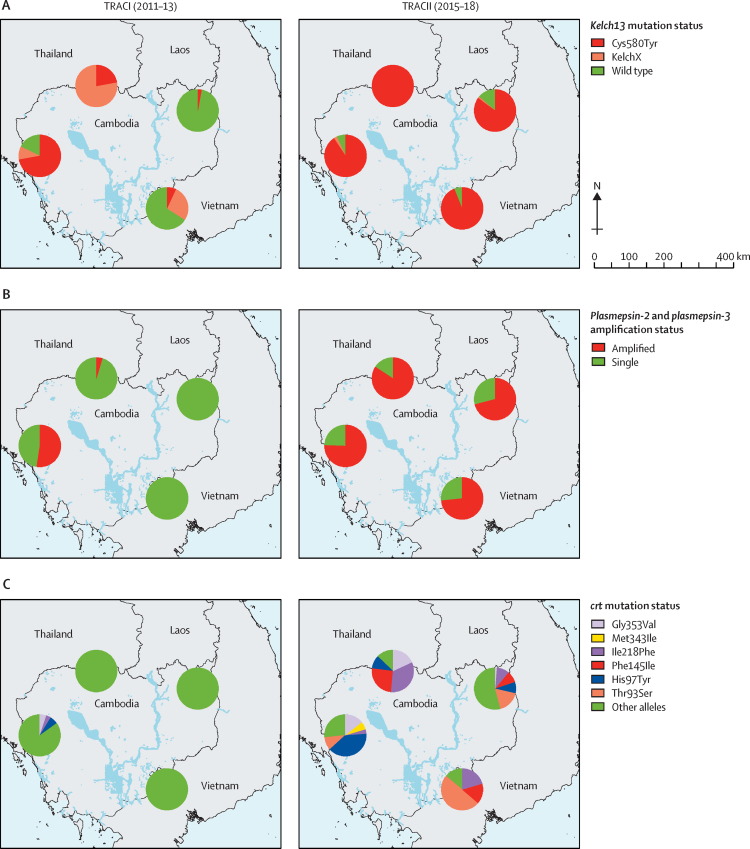
*kelch13* mutation status, *plasmepsin2/3* amplification status, and *crt* mutation status by site and country in the TRACI and TRACII trials (A) *KelchX* mutation status indicates parasites with a *kelch13* mutation other than Cys580Tyr. (B) Single amplification status indicates parasites without a *plasmepsin-2 and plasmepsin-3* amplification. (C) Other *crt* alleles indicate parasites carrying no mutations at positions 93, 97, 145, 218, 343, and 353 of the *crt* gene.

To report the prevalence of molecular markers of resistance, all samples obtained at the study sites during the clinical trial were used, irrespective of the treatment the patient received after enrolment (appendix p 4). We compared the prevalence of molecular markers of resistance in TRACII to the results of our earlier TRACI multicentre study in 2011–13. The marked diversity of *kelch13* mutations in the parasite population previously observed across this region,^4^, ^32^ is now reduced to a predominance of *kelch13* C580Y mutations, present in 369 (91%) of all 404 infections (figure 2; appendix p 9). In TRACI the co-lineage combining the *kelch13* C580Y mutation and the *plasmepsin2/3* amplification was only found in western Cambodia in 72 (43%) of 166 infections, whereas in the current study it was found at all study sites in 296 (73%) of 404 infections. We identified six *crt* mutations that were present in low frequencies in TRACI (prevalence <5·0% in 2011–13). These mutations increased in prevalence between the two studies, appeared to be mutually exclusive, and identified specific haplotypes (appendix p 3, 9). Missingness of genotyping results were mostly caused by low quantities of parasite DNA in the samples or the presence of heterozygote infections.

In the current study, 272 (73%) of 375 parasites carried *crt* mutations at positions 93, 97, 145, 218, 343, or 353. In TRACI only three of these mutations (at positions 97, 218, or 353) were found in 20 (5%) of 368 infections and were present only in western Cambodia. 252 (96%) of the 263 monoclonal infections with these *crt* mutations in the current TRACII study also carried *kelch13* C580Y mutations and 207 (79%) also carried a *plasmepsin2/3* amplification (appendix p 10). Thus, in TRACII 201 (76%) of 263 *crt*-mutated parasites from monoclonal infections carried both a *kelch13* C580Y mutation and had *plasmepsin2/3* amplification. By comparison, during TRACI, 20 (95%) of 21 of *crt*-mutated parasites were also *kelch13* C580Y mutated, and 13 (62%) were *plasmepsin2/3* amplified.

## Discussion

The rapid spread of a co-lineage of multidrug-resistant *P falciparum* across the Greater Mekong subregion has had disastrous consequences for the therapeutic efficacy of dihydroartemisinin-piperaquine in the treatment of uncomplicated *P falciparum* malaria. Alarmingly high rates of treatment failure occurred in Cambodia, Vietnam, and Thailand, which will have contributed to increased transmission of *P falciparum* during the study period. Given its poor efficacy, dihydroartemisinin-piperaquine should no longer be used for the treatment of *P falciparum* malaria in the eastern Greater Mekong subregion. Fortunately, artesunate-mefloquine is known to still be highly effective in these areas,^33^ and Cambodia has now changed its first-line treatment to this ACT. Recent experience^34^ from the Thai-Myanmar border shows that mefloquine resistance can be selected rapidly in the presence of artemisinin resistance, which would likely limit the duration of its efficacy. Worryingly, a recent study^35^ in Preah Vihear (northern Cambodia) showed that the proportion of parasites carrying all three molecular markers of resistance for artemisinins (*kelch13* mutations), piperaquine (*plasmepsin2/3* amplifications), and mefloquine (*mdr1* amplifications) increased from six (7%) of 85 parasites to ten (30%) of 33, after first-line treatment was changed to artesunate-mefloquine in February, 2016. By contrast, our study did not find any parasites carrying both amplifications of *plasmepsin2/3* and *mdr1*. The most recent ACT artesunate-pyronaridine had suboptimal efficacy in curing uncomplicated falciparum malaria in western Cambodia in 2007–08 and 2014–15,^36^, ^37^ but more recent assessments in eastern Cambodia showed adequate cure rates.^38^ Artesunate-pyronaridine has now been proposed to replace dihydroartemisinin-piperaquine in northeastern Thailand and southern Vietnam.

Patients often presented with gametocytaemia and detectable plasma piperaquine at the moment of enrolment, which suggests that these patients had a recrudescent rather than a primary infection. An association between gametocytaemia and recrudescent infections was also observed when mefloquine resistance emerged in the 1990s on the western border of Thailand,^39^ and is likely to promote the spread of drug resistance. In our study, gametocytes were detected by microscopy in only one of 65 patients at recrudescence, which might be explained by the early detection at low parasitaemias in the study setting, before late-stage gametocytes become visible in peripheral blood. Additionally, the use of gametocytocidal primaquine at baseline will have shortened gametocyte carriage.

Over-representation of potential recrudescent infections on enrolment could have resulted in an underestimation of the efficacy of dihydroartemisinin-piperaquine in primary infections. However, the combination of high treatment coverage with the first-line treatment dihydroartemisinin-piperaquine, and high treatment failure in the same area causes an increasing number of infections to be recrudescences, which will contribute importantly to the malaria burden. Low malaria transmission in the study area precluded a larger sample size. The sample size in Western Cambodia is smaller because the antimalarial drug in one study group was changed from dihydroartemisinin-piperaquine to artesunate-mefloquine. The results reported from geographically diverse sites in the study area are representative for the eastern Greater Mekong subregion.

The multidrug-resistant parasite co-lineage that originated in western Cambodia over 10 years ago has spread and evolved.^25^ Day 7 piperaquine concentrations were not predictive of treatment failure in this study, whereas they are in piperaquine-sensitive infections.^31^ This finding strongly suggests that in the current study parasite resistance rather than reduced exposure to piperaquine was the main determinant of treatment failure. The patient showing very early treatment failure apparent at day 9 after treatment was infected with parasites containing a *kelch13* C580Y mutation, *plasmepsin2/3* amplification, and a *crt* T93S mutation. However, the plasma piperaquine concentration at the time of recrudescense was low with 15·1 ng/mL, indicating possible underexposure to piperaquine that could have contributed to this very early treatment failure.

In addition to the previously defined marker of piperaquine resistance, amplification of *plasmepsin2/3*, we found that mutations in the *crt* gene were also associated with treatment failure after dihydroartemisinin-piperaquine. Most *crt* mutations described in this study (H97Y, F145I, and G353V) have also been identified in recent gene-editing experiments by Ross and colleagues^18^ to confer piperaquine resistance in vitro. They showed that all mutations except the *crt* Met343Leu (M343L) mutation resulted in a fitness loss. We did not observe clear selection of the *crt* M343L mutation, which can be related to the difference in determinants governing parasite fitness in the field setting compared with determinants obtainable in the laboratory, including the increased transmissibility of ineffectively treated infections. The findings in our study, in combination with the findings of Ross and colleagues^18^ support the hypothesis that the *crt* mutations affect parasite sensitivity to piperaquine. Most *crt* mutations occurred in parasites carrying the *kelch13* C580Y mutation and *plasmepsin2/3* amplification. These *crt* mutations are distinct from the known *crt* variants conferring chloroquine resistance.

A major concern is that artemisinin and partner drug resistance will continue to evolve, producing parasite strains more capable of surviving treatment, which can subsequently spread across a wider geographical area. Resistance to chloroquine and sulphadoxine-pyrimethamine has emerged in the Greater Mekong subregion in the past, and subsequently migrated from southeast Asia to the Indian subcontinent and sub-Saharan Africa, likely contributing to millions of deaths from severe malaria in African children.^40^, ^41^ In the absence of new drug classes to replace current first-line therapies, the use of existing drugs in the form of triple ACTs, in which an artemisinin component is combined with two partner drugs, could be a viable alternative. The results of the TRACII study are expected to be published later this year. The preliminary results indicated dihydroartemisinin-piperaquine and mefloquine to be fully efficacious in Thailand, Cambodia, and Vietnam. Nevertheless, accelerated elimination of all *P falciparum* in the Greater Mekong subregion is needed urgently.^42^ Expansion and further spread of very difficult to treat, highly resistant *P falciparum* would cause a regional and potentially global health emergency.
