# Supplementary material for: Determinants of dihydroartemisinin-piperaquine treatment failure in Plasmodium falciparum malaria in Cambodia, Thailand, and Vietnam: a prospective clinical, pharmacological, and genetic study
Source: Lancet Infect Dis. 2019 Sep;19(9):952–61. doi: 10.1016/S1473-3099(19)30391-3 (PMC6715822; doi:10.1016/S1473-3099(19)30391-3)
Supplement: Supplementary appendix [file mmc1.pdf]

THE LANCET Infectious Diseases

Supplementary webappendix

This webappendix formed part of the original submission and has been peer reviewed. We post it as supplied by the authors.

Supplement to: van der Pluijm RW, Imwong M, Chau NH, et al. Determinants of dihydroartemisinin-piperaquine treatment failure in *Plasmodium falciparum* malaria in Cambodia, Thailand, and Vietnam: a prospective clinical, pharmacological, and genetic study. *Lancet Infect Dis* 2019; published online July 22. http://dx.doi.org/10.1016/S1473-3099(19)30391-3.

Appendix

Table S1. Predictors of treatment failures				
	HR (unadjusted) (95% CI)	p-value	HR (adjusted) (95% CI)	p-value
**Site**				
Ratanakiri, Cambodia (n=44)	1·000			
Binh Phuoc, Vietnam (n=60)	2·591 (1·293-5·192)	0·007		
Western Cambodia (n=17)	2·828 (1·198-6·676)	0·018		
North-eastern Thailand (n=19)	6·194 (2·821-13·600)	<0·001		
**Sex**				
Female (n=22)	1·000			
Male (n=118)	1·880 (0·857-4·123)	0·115		
**Age (year)**	0·993 (0·972-1·014)	0·492		
**Baseline parasite count**	1·000 (0·999-1·000)	0.064		
**Parasite clearance half-life**	1·166 (1·018-1·335)	0·027		
**Piperaquine measurable at baseline**				
No (n=101)	1·000			
Yes (n=30)	1·695 (0·978-2·937)	0·060		
***Kelch13* status**				
WT (n=12)	1·000		1·000	
C580Y (n=119)	3·863 (0·943-15·816)	0·060	0·632 (0·125-3·208)	0·580
***Plasmepsin2/3* amplification status**				
No amplification (n=36)	1·000		1·000	
Amplification (n=103)	4·619 (1·991-10·717)	<0·001	3·200 (1·279-8·008)	0·013
**Piperaquine levels at day 7 (continuous)**	1·003 (0·998-1·009)	0·186		
Piperaquine levels at day 7>=30ng/ml (n=72)	1.000			
Piperaquine levels at day 7<30ng/ml (n=53)	0·991 (0·581-1·690)	0.974		
***CRT* mutation status**				
Other *crt* alleles (n=32)	1·000		1·000	
Thr93Ser (n=31)	3·880 (1·617-9·313)	0·002	4·539 (1·562-13·185)	0·005
His97Tyr (n=15)	3·017 (1·057-8·613)	0·039	3·841 (1·193-12·368)	0·024
Phe145Ile (n=16)	4·942 (1·875-13·029)	0·001	7·541 (2·396-23·732)	0·001
Ile218Phe (n=25)	3·996 (1·608-9·931)	0·003	5·099 (1·715-15·161)	0·003
Met343Ile (n=2)	5·413 (1·116-26·251)	*	5·831 (1·074-31·663)	*
Gly353Val (n=3)	8·165 (2·094-31·835)	*	10·475 (1·931-56·821)	*
*Statistical significance not assessed due to small numbers. HR=Hazard Ratio. WT=Wild type.				

Targets	Chromosome	Start	Stop	Length	Multiplex	Forward Primer Sequence	Reverse Primer Sequence
CRT 72, 74, 75, 76	Pf3D7_07_v3	403483	403687	204	1	ACACTCTTTCCCTACACGACGCTCTTCCG ATCTTTAACAGATGGCTCACGTTmUA	TCGGCATTCCTGCTGAACCGCTCTTCCGA TCTGAGTTTCGGATGTTACAAAAmCT
CRT 93, 97	Pf3D7_07_v3	403629	403818	189	2	ACACTCTTTCCCTACACGACGCTCTTCCG ATCTTTTTGCTAAAAGAACTTTAAAmCA	TCGGCATTCCTGCTGAACCGCTCTTCCGA TCTTTGGTAGGTGGAATAGATTCmUC
CRT 218, 220	Pf3D7_07_v3	404352	404600	248	1	ACACTCTTTCCCTACACGACGCTCTTCCG ATCTATCTTTTGAAACACAAGAAGmAA	TCGGCATTCCTGCTGAACCGCTCTTCCGA TCTATTTCCCTTGTCATGTTTGAmAA
CRT 271	Pf3D7_07_v3	404778	405026	248	2	ACACTCTTTCCCTACACGACGCTCTTCCG ATCTTTTCCAATTGTTCACTTCTTmGT	TCGGCATTCCTGCTGAACCGCTCTTCCGA TCTTATTTTACCTCTACGACTGTmGT
CRT 326, 333	Pf3D7_07_v3	405189	405432	243	1	ACACTCTTTCCCTACACGACGCTCTTCCG ATCTGAGCATGGGTAAGAAGCTTAmUA	TCGGCATTCCTGCTGAACCGCTCTTCCGA TCTCCTCTTGTATGTATCAACGTmUT
CRT 356	Pf3D7_07_v3	405574	405763	189	2	ACACTCTTTCCCTACACGACGCTCTTCCG ATCTTGTTAGTTGTATACAAGGTCmCA	TCGGCATTCCTGCTGAACCGCTCTTCCGA TCTACGTTGTACCATCATAAACAmUT
CRT 371	Pf3D7_07_v3	405753	405965	212	1	ACACTCTTTCCCTACACGACGCTCTTCCG ATCTTGGTACAACGTATCATATTTmUA	TCGGCATTCCTGCTGAACCGCTCTTCCGA TCTACGAACAAGCCATTTGATATmUA
kelch13 BTB/POZ	Pf3D7_13_v3	1724912	1725156	244	1	ACACTCTTTCCCTACACGACGCTCTTCCG ATCTATGAATTTAGAACTTCGCCAmUT	TCGGCATTCCTGCTGAACCGCTCTTCCGA TCTTCCATATGCCTTATTAGAAGmCT
kelch13 BTB/POZ	Pf3D7_13_v3	1725070	1725319	249	2	ACACTCTTTCCCTACACGACGCTCTTCCG ATCTTCATTATCAATACCTCCAACAmAC	TCGGCATTCCTGCTGAACCGCTCTTCCGA TCTATCGTATGAAAGCATGGGTmAG
kelch13 BTB/POZ	Pf3D7_13_v3	1725261	1725475	214	1	ACACTCTTTCCCTACACGACGCTCTTCCG ATCTCATAGCTGATGATCTAGGmGG	TCGGCATTCCTGCTGAACCGCTCTTCCGA TCTCTGAGGTGTATGATCGTTTAmAG
kelch13 BTB/POZ	Pf3D7_13_v3	1725428	1725657	229	2	ACACTCTTTCCCTACACGACGCTCTTCCG ATCTAATTACTTGAAACATACCATmAC	TCGGCATTCCTGCTGAACCGCTCTTCCGA TCTTATAGGTGGATTTGATGGTGmUA
kelch13 BTB/POZ	Pf3D7_13_v3	1725566	1725814	248	1	ACACTCTTTCCCTACACGACGCTCTTCCG ATCTTAGACATAGGTGTACACATAmCG	TCGGCATTCCTGCTGAACCGCTCTTCCGA TCTTCTTAGATAGGGATAGTGAGmUT
kelch13 BTB/POZ	Pf3D7_13_v3	1725746	1725980	234	2	ACACTCTTTCCCTACACGACGCTCTTCCG ATCTTGGGTATAGTTAACGGATTTmCT	TCGGCATTCCTGCTGAACCGCTCTTCCGA TCTAAAATTGTTGATGCAAATATmUG
Plasmepsin 2/3 breakpoint	Pf3D7_14_v3	298737	289836	var	2	ACACTCTTTCCCTACACGACGCTCTTCCG ATCTCTAGGTGACCCATTTATGmAG	TCGGCATTCCTGCTGAACCGCTCTTCCGA TCTTAGCTTTAGCATCATTCAmCG

Table S2 – Amplicon sequencing primers used to genotype variations in the *crt, kelch13* and *plasmepsin2/3* genes.
In order avoid amplicon overlapping, two different multiplexes were designed. For each amplicon, we show, from left to right: the target variants located within the amplicon, the chromosome, start and end location of the amplicon (using the Pf3D7_v3 reference); the length of the amplicon; the multiplex within which the amplicon was implemented; and the forward and reverse primer sequences.

CRT Haplotype	Counts		Allele at individual positions of the *crt* gene															
	TRACI	TRACII	72-76	220	271	371	326	356	93	97	145	218	343	353	144	148	194	333
Wild-type (3D7-like)	9	0	CVMNK	A	Q	R	N	I	T	H	F	I	M	G	A	L	I	T
CVIET	38	0	CVIET	S	E	I	N	I	T	H	F	I	M	G	A	L	I	T
CVIET+Ile194Thr	10	0	CVIET	S	E	I	N	I	T	H	F	I	M	G	A	L	T/(-)	T
CVIET+Ile356Thr	1	0	CVIET	S	E	I	N	T	T	H	F	I	M	G	A	L	I	T
CVIET+N326S+356Thr	191	50	CVIET	S	E	I	S	T	T	H	F	I	M	G	A	L	I/(-)	T
Thr93Ser mutation	0	82	CVIET	S	E	I	S	T	S	H	F	I	M	G	A	L	I	T
His97Tyr mutation	10	69	CVIET	S	E	I	S	T	T	Y	F	I	M	G	A	L	I	T
Phe145Ile mutation	0	35	CVIET	S	E	I	S	T	T	H	I	I	M	G	A	L	I	T
Ile218Phe mutation	6	48	CVIET	S	E	I	S	T	T	H	F	F	M	G	A	L	I	T
Met343L mutation	2	0	CVIET	S	E	I	S	T	T	H	F	I	L	G	A	L	I	T
Met343Ile mutation	0	10	CVIET	S	E	I	S	T	T	H	F	I	I	G	A	L	I	T
Gly353Val mutation	8	29	CVIET	S	E	I	S	T	T	H	F	I	M	V	A	L	I	T
CVIDT+Thr333Ser	49	4	CVIDT	S	E	R	N	I	T	H	F	I	M	G	F	I	T/(-)	S
CVIDT+Arg371Ile	1	0	CVIDT	S	E	I	N	I	T	H	F	I	M	G	F	I	T	T
Heterozygote infections	49	50																
Missingness at position 93, 97, 145, 218, 343 or 353	48	7																
Haplotyping not possible	6	47																

Table S3 – Prevalence of *crt* gene haplotypes in TRACI (2011-2013) and TRACII (2015-2018).

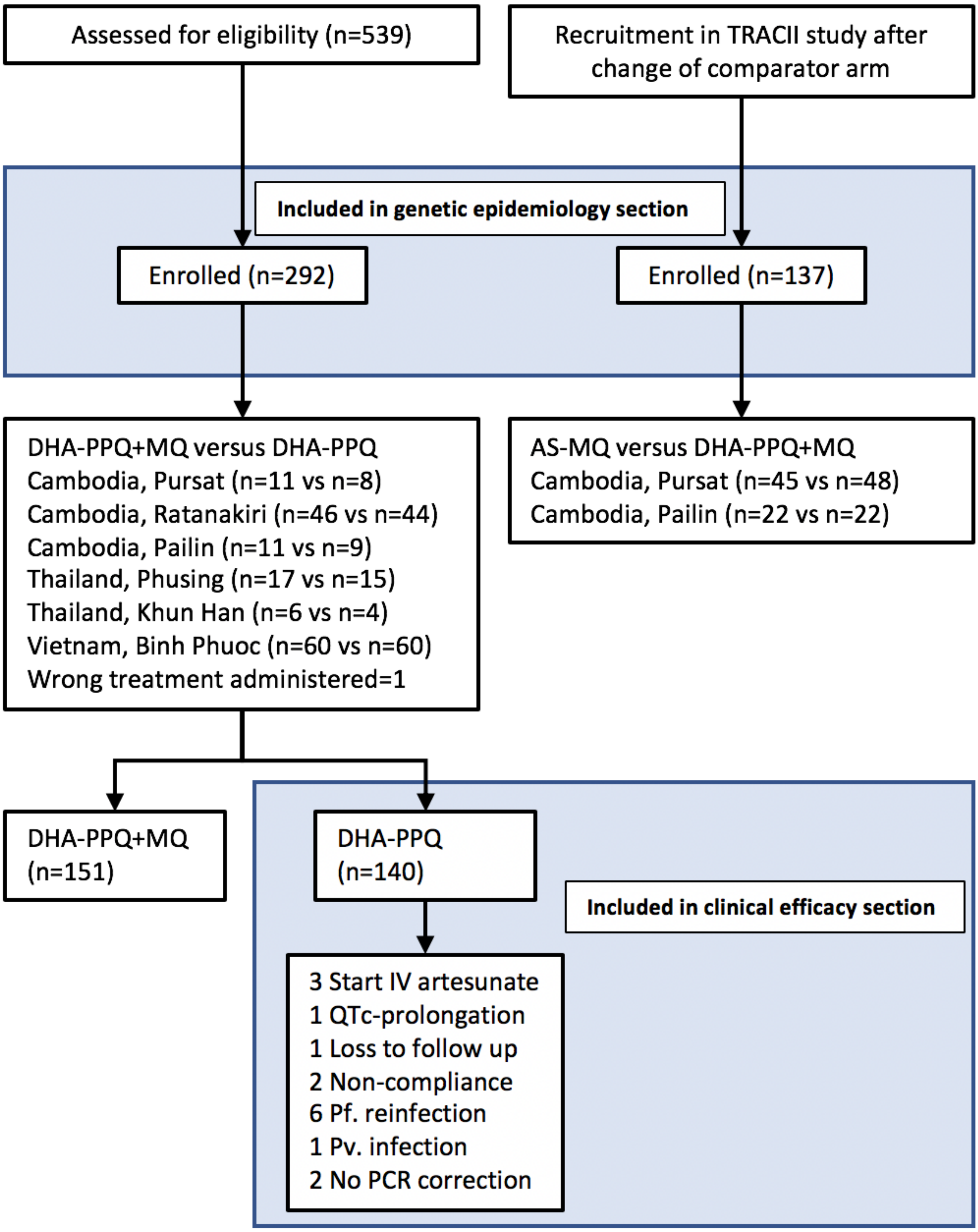

Figure S1. Simplified flow diagram of the TRACII study. The two panels indicate which patients have been included in the different sections that are presented in this manuscript.

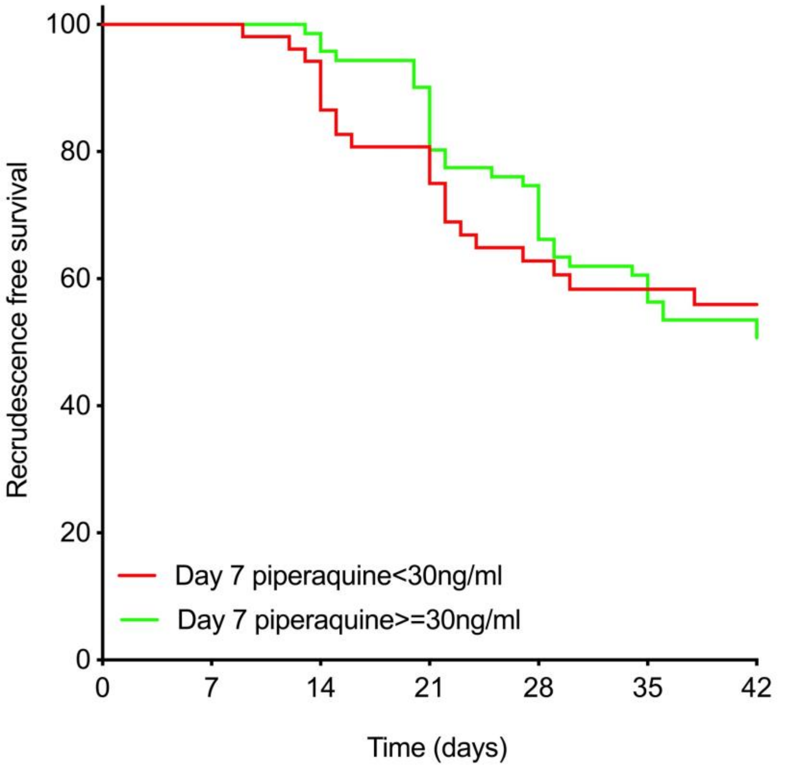

Number at risk

Day 7 piperaquine<30ng/ml	53	52	49	42	30	26	23
Day 7 piperaquine>=30ng/ml	72	71	70	64	52	43	38

Figure S2. Kaplan-Meier survival curves describing PCR corrected efficacy of dihydroartemisin-piperaquine by day 7 piperaquine concentrations.

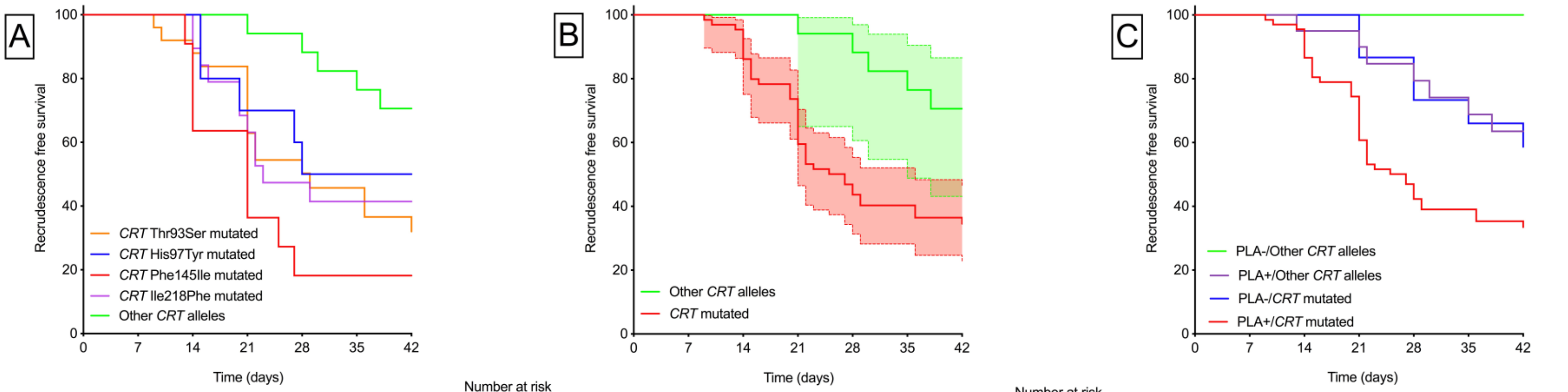

Number at risk

CRT Thr93Ser mutated	26	25	23	20	13	10	8
PfCRT His97Tyr mutated	10	10	10	7	6	5	4
PfCRT Phe145Ile mutated	11	11	10	7	2	2	2
PfCRT Ile218Phe mutated	20	19	19	13	8	7	5
Other *CRT* alleles	17	17	17	17	16	14	12

Number at risk

Other *CRT* alleles	17	17	17	17	16	14	12
CRT mutated	67	65	62	47	29	24	19

Number at risk

PLA-/Other *CRT* alleles	12	12	12	12	12	12	12
PLA+/Other *CRT* alleles	20	20	19	19	16	14	12
PLA-/*CRT* mutated	18	15	15	15	12	10	9
PLA+/*CRT* mutated	69	67	64	49	30	24	19

Figure S3. Kaplan-Meier survival curves describing PCR corrected efficacy of dihydroartemisin-piperaquine by *crt* mutation status (for the subgroup of parasites bearing a *Kelch13* Cys580Tyr mutation and *Plasmepsin2/3* amplification) (Panel A and B) and a combination of *Plasmepsin2/3* amplification and *crt* mutation status (Panel C). Panel A/B/C: ‘Other *crt* alleles’ indicates parasites carrying no mutations at position 93, 97 and 145, 218, 343 and 353 of the *crt* gene. Panel B: Shaded areas indicate 95 % confidence intervals. Panel C: **PLA+** indicates parasites carrying a *Plasmepsin2/3* amplification. **PLA-** indicates parasites carrying a single copy of *Plasmepsin2/3*. Panel B/C: Dotted lines indicated 95 % confidence intervals.

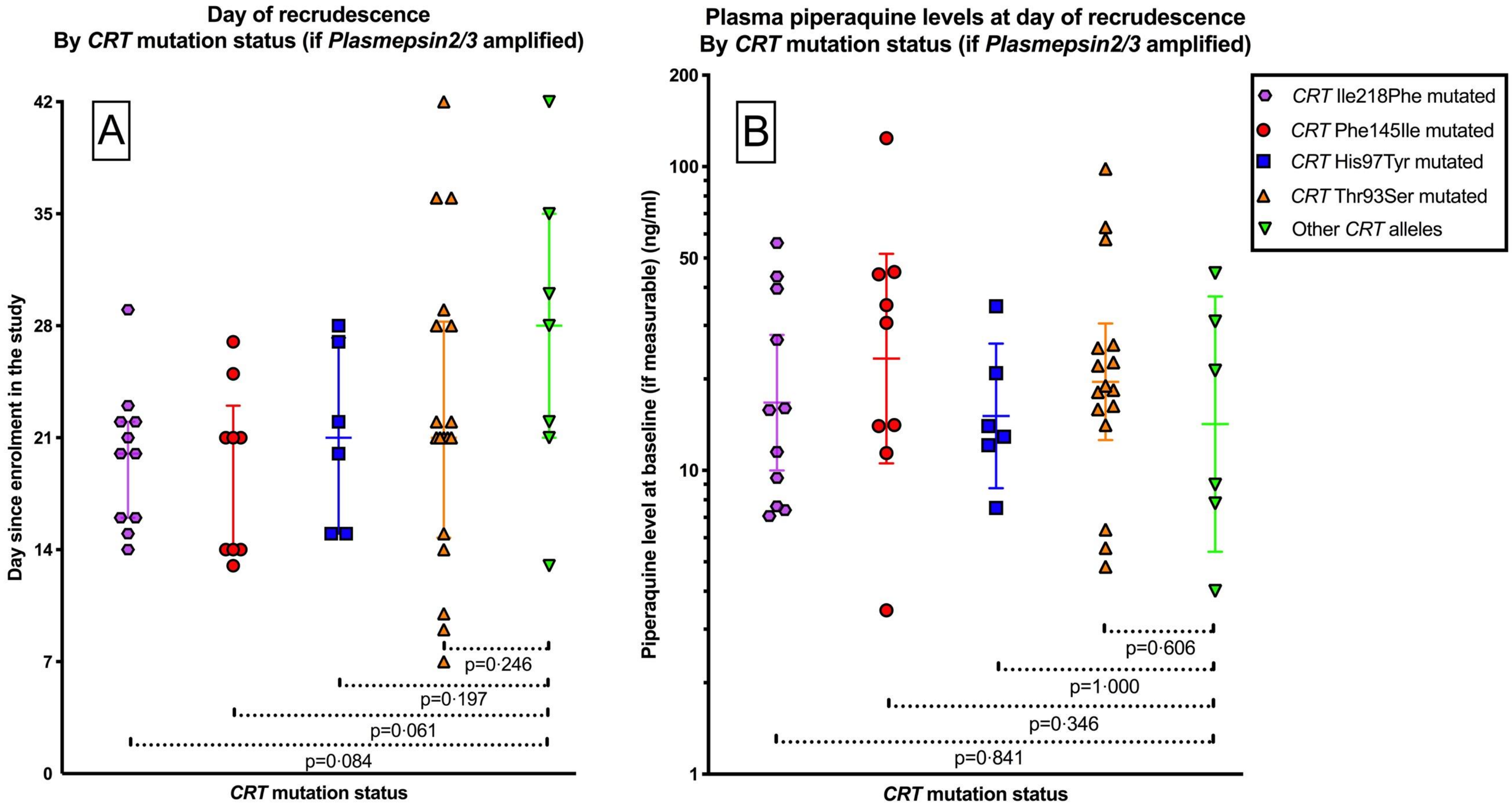

Figure S4. Day of recrudescence (Panel A) and piperaquine levels at day of recrudescence (Panel B) by *crt* mutation status for subgroup of parasites with a *Plasmepsin2/3* amplification. Panel A: Bars indicate median and interquartile ranges. Panel B: Bars indicate geometric mean and 95% confidence intervals.

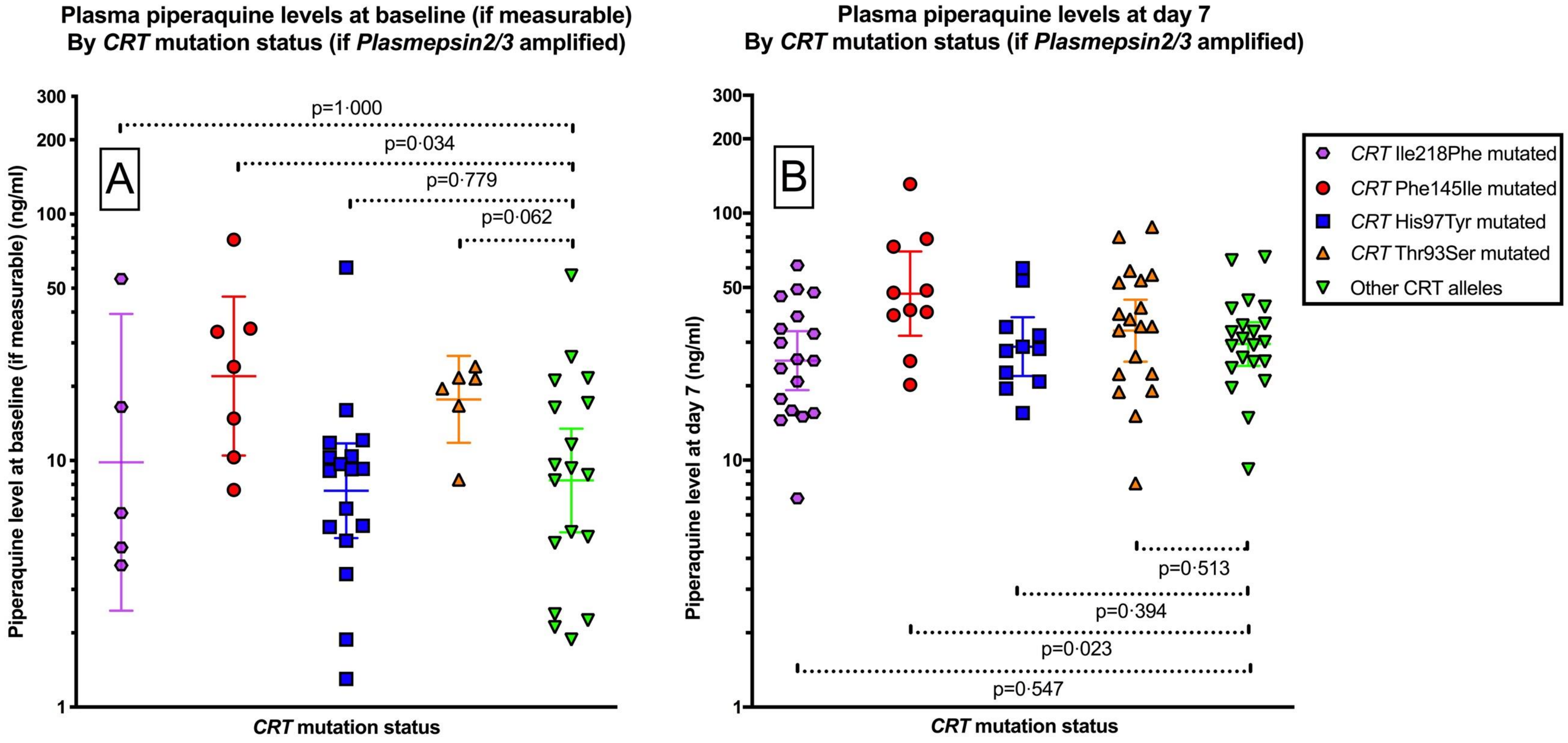

Figure S5. Baseline piperaquine levels (Panel A) and piperaquine levels at day 7 (Panel B) by *crt* mutation status for subgroup of parasites with a *Plasmepsin2/3* amplification. Bars indicate geometric mean and 95% confidence intervals.

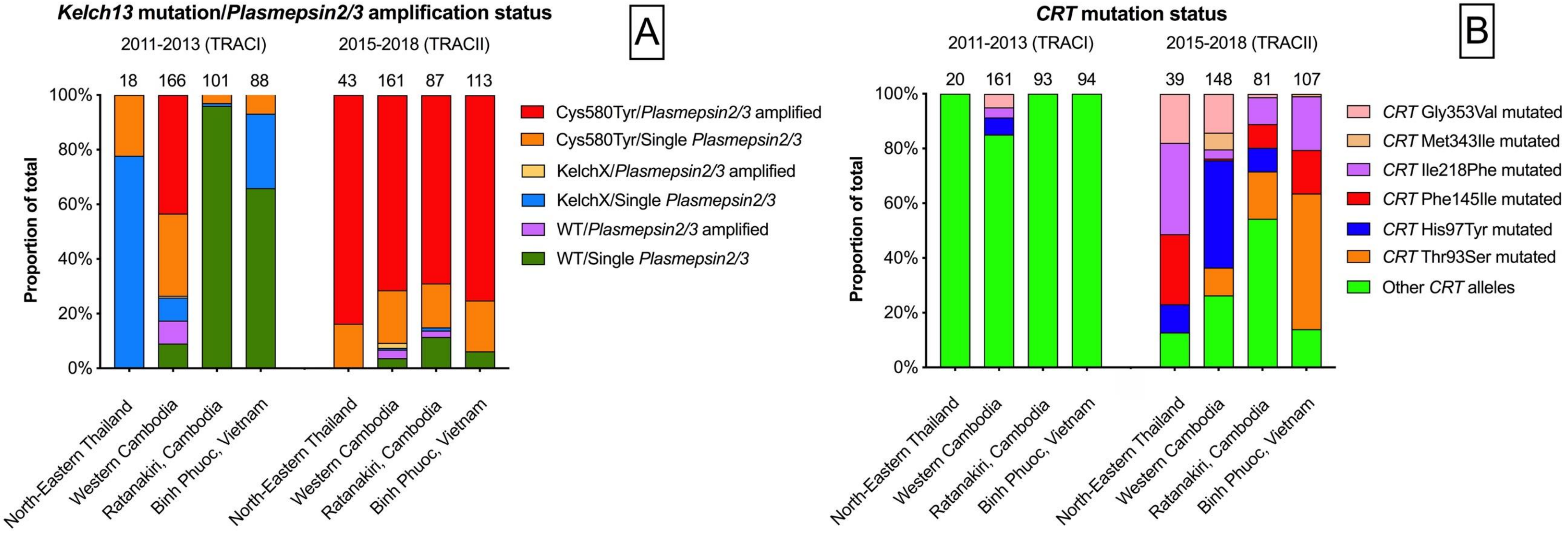

Figure S6. Frequencies of combinations for genetic markers relevant to resistance to artemisinins and piperaquine at the four sites/regions in TRACI (2011-2013) and TRACII (2015-2018). Panel A: **Cys580Tyr, KelchX and WT** indicate a *Kelch13* Cys580Tyr mutation, a *Kelch13* other than Cys580Tyr and *Kelch13* wild-type, respectively. '***Plasmepsin2/3* amplified'** and **'Single *Plasmepsin2/3'*** indicate parasites with or without a *Plasmepsin2/3* amplification, respectively. Panel B: Prevalence of *crt* **Thr93Ser, His97Tyr, Phe145Ile, Ile218Phe, Met343Ile and Gly353Val** mutations. '**Other *CRT* alleles'** indicates parasites carrying no mutations at position 93, 97 and 145, 218, 343 and 353 of the *crt* gene.

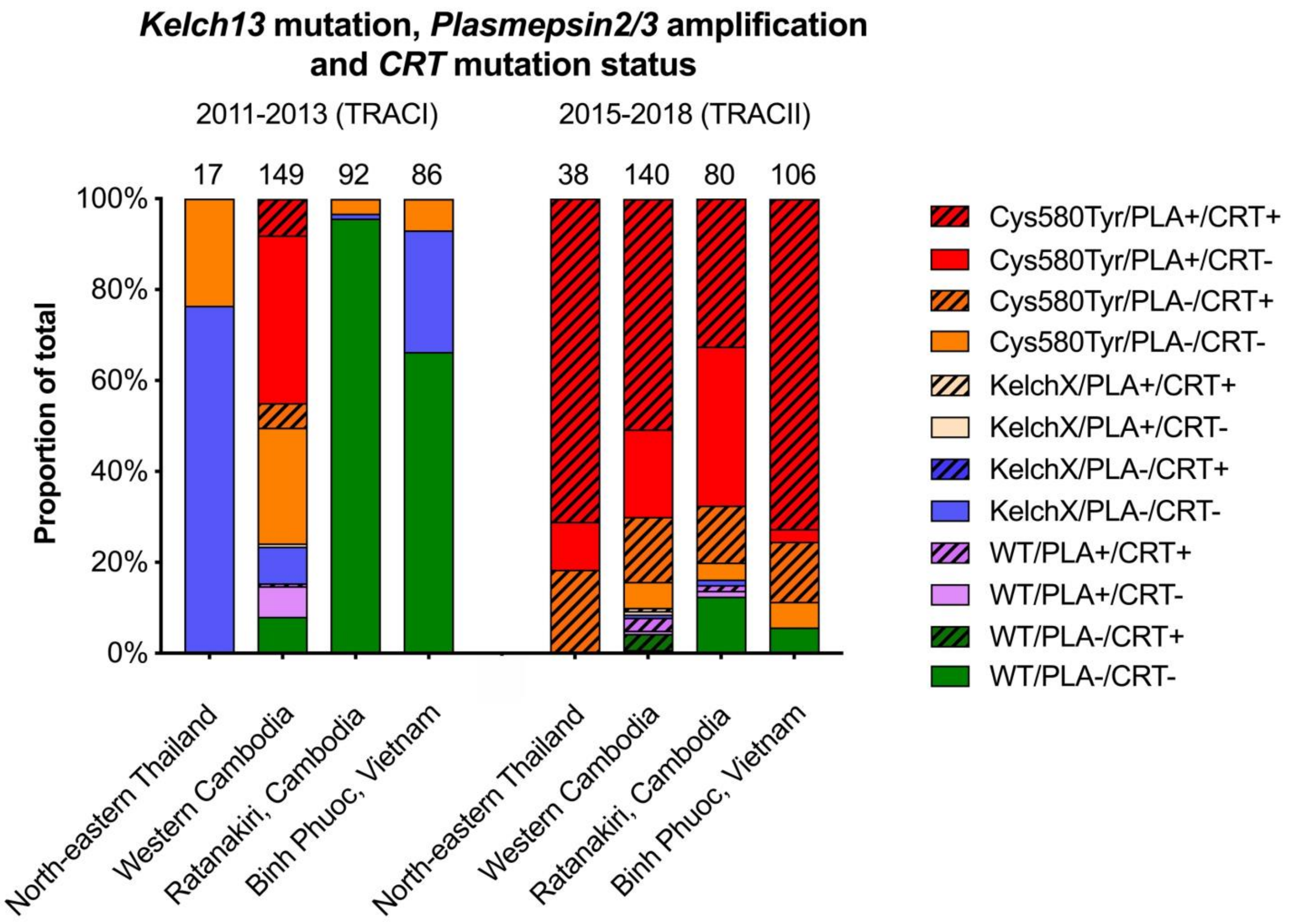

Figure S7. Frequencies of combinations for genetic markers related to resistance to artemisinins and piperaquine in TRACI (2011-2013) and TRACII (2015-2018). **Cys580Tyr, KelchX and WT** indicate a *Kelch13* Cys580Tyr mutation, a *Kelch13* other than Cys580Tyr and *Kelch13* wild-type, respectively. **'PLA+'** and **'PLA-'** indicate parasites with or without a *Plasmepsin2/3* amplification, respectively. **'CRT+'** indicate parasites with one of the *crt* **Thr93Ser, His97Tyr, Phe145Ile, Ile218Phe, Met343Ile and Gly353Val** mutations whereas **'CRT-'** identifies parasites without of one of these mutations.

Details on medication used in trial:

DHA-piperaquine dosing schedule (administered at H0, H24 and H48)

DHA-piperaquine	
**Weight (kilogram)**	**Tablets/day (40/320 mg/tablet)**
**5 – 7·9**	**0·5**
**8 - 10·9**	**0·75**
**11 – 16·9**	**1**
**17 – 24·9**	**1·5**
**25 - 35·9**	**2**
**36 - 59·9**	**3**
**60 - 80·9**	**4**
**81 and more**	**5**

DHA-piperaquine formulations and sourcing

Vietnam:	Arterakine	Drug Company Central 1, Vietnam	Batch: 12010/13002
Cambodia:	D-Artepp	Guilin Pharmaceutical, China	Batch: SQ150501
Thailand:	D-Artepp	Guilin Pharmaceutical, China	Batch: SQ150501/SQ160438

Primaquine treatment schedule (administered at H24)

Primaquine	
**Weight (kilogram)**	**Tablet/day (15 mg/tablet)**
**<25**	**0·25**
**25-50**	**0·50**
**>50**	**1·00**

Primaquine formulations and sourcing

Vietnam:	Primaquine	Danapha, Vietnam	Batch: 10815
Thailand:	Primaquine	GPO, Thailand	Batch: L580200/L580466
Cambodia:	Primaquine	GPO, Thailand	Batch: 64329

Mefloquine dosing schedule (administered at H0, H24 and H48)

Mefloquine	
**Weight (kilogram)**	**Milliliter/day (50mg/ml)**
5 - 5.9	0.8
6 - 6.9	1
7 - 7.9	1.2
8 - 8.9	1.3
9 - 9.9	1.5
10 - 10.9	1.7
11 - 11.9	1.8
**Weight (kilogram)**	**Tablet/day (250 mg/tablet)**
12 - 16.9	0.5
17 - 23.9	0.75
24 - 24.9	1
25 - 33.9	1
34 - 35.9	1.25
36 - 43.9	1.25
44 - 48.9	1.5
49 - 53.9	1.75
54 - 59.9	2
60 - 63.9	2
64 - 71.9	2.25
72 - 77.9	2.5
78 - 80.9	2.75
81 and more	2.75

Mefloquine:
Note: For younger children ($\leq$ 11 kg), mefloquine can be given by dissolving in water or other beverage (e.g. fruit juice) and a suspension is made by allowing 1 tablet to dissolve in 5ml (1ml=50mg)

Mefloquine formulations and sourcing

Vietnam:	Lariam	Roche Pharmaceuticals, Switzerland	Batch: B1220B02
Cambodia:	Lariam	Roche Pharmaceuticals, Switzerland	Batch: B1218B02
Thailand:	Mequin	Atlantic Laboratories Ltd, Thailand	Batch: 140130

Treatment of recurrent infections

Cambodia/Thailand   Artesunate+atovaquone-proguanil for 3 days

Artesunate dosing schedule

Artesunate			
Weight (kilogram)	Tablets/day (50mg/tablet)	Weight (kilogram)	Tablets/day (50mg/tablet)
11-14·9	1	43-45·9	3·5
15-16·9	1·25	46-48·9	3·75
17-20·9	1·5	49-51·9	4
21-23·9	1·75	52-54·9	4·25
24-26·9	2	55-57·9	4·5
27-29·9	2·25	58-60·9	4·75
30-32·9	2·5	61-64·9	5
33-35·9	2·75	65-67·9	5·25
36-39·9	3	68 and above	5·5
40-42·9	3·25		

Artesunate formulation and sourcing

Cambodia	Artesunate	Guilin Pharmaceutical, China	Batch: B1228B07
Thailand	Artesunate	Guilin Pharmaceutical, China	Batch: AS140807

Atovaquone-proguanil dosing schedule

Atovaquone-proguanil	
Weight (kilogram)	Tablets/day (250-100 mg/tablet)
11-20·9 kg	1
21 – 30·9	2
31 – 39·9	3
40 and above	4

Atovaquone-proguanil formulation and sourcing

Cambodia	Malanil (250-100 mg)	GSK, United Kingdom	Batch: 2G005
Thailand	Malanil (250-100 mg)	GSK, United Kingdom	Batch: 2G005/C3001

**Vietnam**	**Quinine+doxycycline for 7 days**		

Quinine dosing schedule

Quinine (30 mg/kg/day t.i.d.)

Quinine formulation and sourcing

Vietnam:	Quinine	GPO, Thailand	Batch: F571314

Doxycycline dosing schedule

Doxycycline (3mg/kg/day q.d)

Doxycycline formulation and sourcing

Vietnam:	Doxycycline	STADA-VN, Vietnam	Batch: 051215
